# The Role of Micrornas in The Development of Hospital Acquired Infection in Polytrauma Patients

**DOI:** 10.1186/2197-425X-3-S1-A35

**Published:** 2015-10-01

**Authors:** HC Owen, HDT Torrance, MR Barnes, K Brohi, JC Knight, CJ Hinds, MJ O'Dwyer

**Affiliations:** Barts & the London School of Medicine, QMUL, Centre for Translational Medicine and Therapeutics, William Harvey Research Institute, London, United Kingdom; Barts Health NHS Trust, Adult Critical Care Unit, Royal London Hospital, London, United Kingdom; Barts & the London School of Medicine, QMUL, Centre for Trauma Sciences, Blizard Institute, London, United Kingdom; Barts & the London School of Medicine, QMUL, William Harvey Research Institute, London, United Kingdom; University of Oxford, Wellcome Trust Centre for Human Genetics, Oxford, United Kingdom

## Introduction

Traumatic injury is associated with immunosuppression and an increased risk of developing nosocomial infections. However, the immune regulatory mechanisms involved remain unclear.

## Objectives

1) To describe genome-wide alterations in micro RNA (miRNA) expression following severe trauma.

2) To explore the potential role of miRNAs in mediating the post-traumatic immunosuppressive phenotype and their potential role in enhancing the risk of nosocomial infections.

## Methods

Patients requiring ICU care following traumatic injury were recruited. Whole blood was collected within 2 hours of injury and 24 hours later. Total RNA (containing miRNAs) was isolated utilising PAX Gene and RNA extraction kits (Qiagen). miRNA-sequencing was performed with the Illumina HiSeq2500, and sequences were aligned to the human GRCh37 reference genome. Data analysis was carried out using the DESEQ2 package in R, and miRNAs were considered significantly altered with an adjusted p value of < 0.05. Functional enrichment analysis was performed using Ingenuity Pathway Analysis (IPA) on all miRNAs reaching an adjusted p value of < 0.1. mRNA targets of interest were identified using miRBase and TargetScan (http://www.mirbase.org, http://www.targetscan.org).

## Results

**Figure 2:**
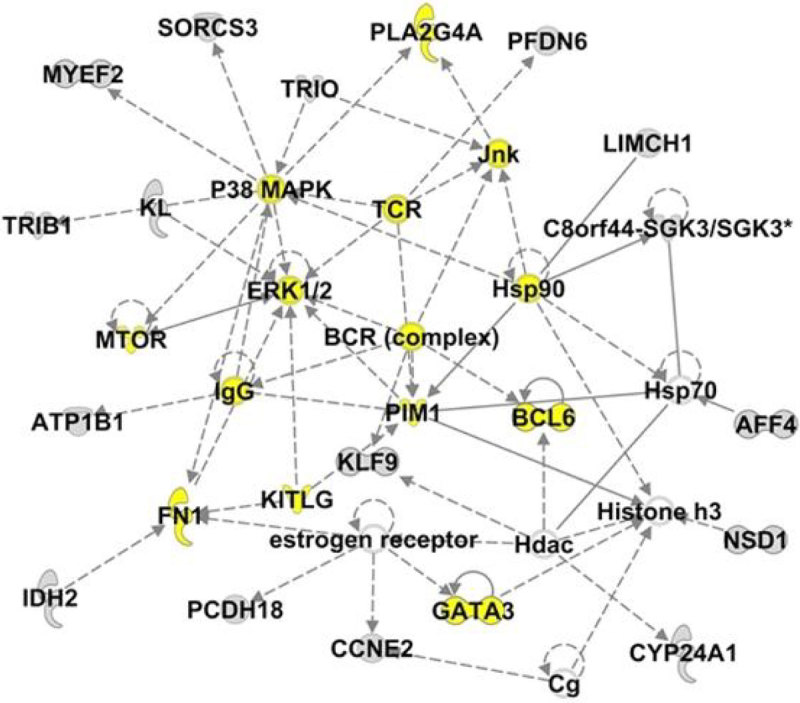
**miR-144 mRNA targets create networks linked to the cell-mediated immune response. Genes linked to the immune response are highlighted in yellow.**

49 patients were recruited and 25 patients developed nosocomial infections. Expression of 139 miRNAs was significantly altered between 2 hours and 24 hours following injury, with miR-146b, a key inhibitor of pro-inflammatory pathways[1], upregulated to the greatest degree. Figure 1 presents miRNAs that differ between those patients who developed nosocomial infections and those who did not. miR-144-5p was significantly different between the two groups at both time points. a large percentage of mRNA targets for miR-144 are involved the Cell-mediated Immune Response (Figure 2), including the B-cell receptor complex, p38MAPK, GATA3, IgG, BCL6 and the T-cell receptor. in addition, we have previously shown that the miR-374 family of miRNAs is linked to increased IL-10 expression in trauma patients[2]. IPA highlights Cancer, Haematological Disease, Immunological and Inflammatory Disease and Organismal Injury and Abnormalities as important pathways altered between infected and non-infected patients.

**Figure:**
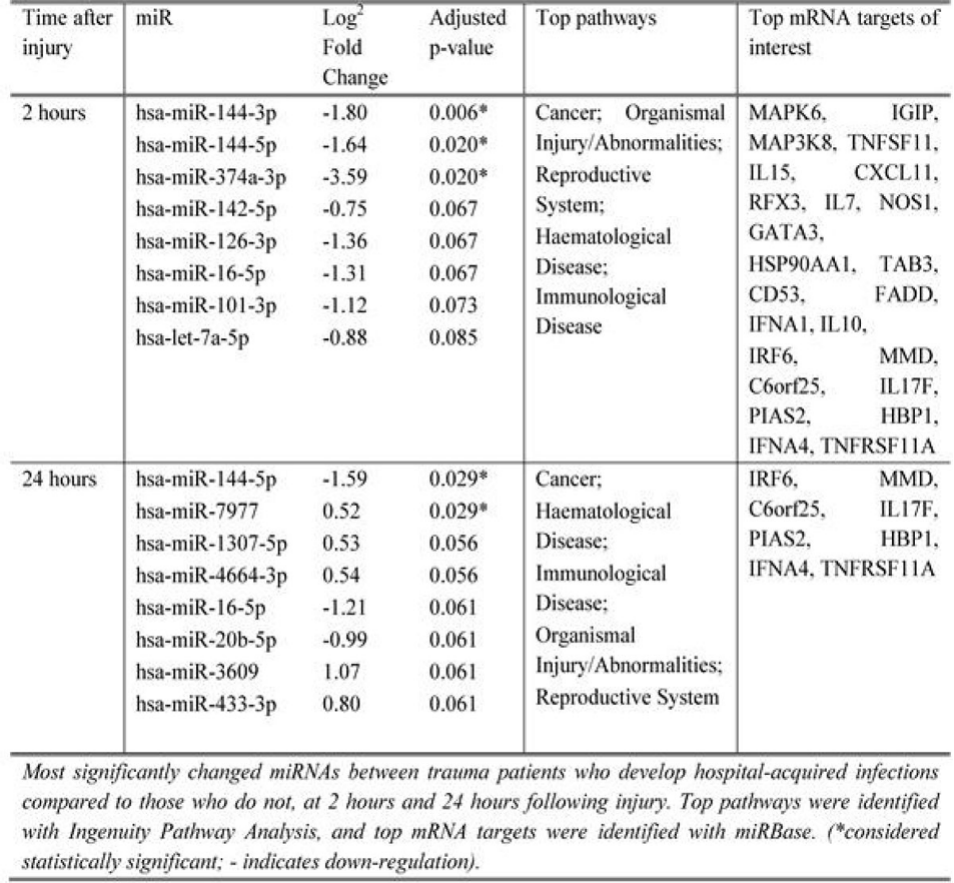
Figure 1

## Conclusions

These data provide a miRNA signature of severely injured trauma patients who develop hospital acquired infection compared to those who do not, and identify the miR-144 and miR-374b families as being of particular interest for future studies of trauma-induced immune dysfunction.

## Grant Acknowledgment

This work was funded by a Barts and the London Charity Grant.
